# Factors Relating to Sprint Swimming Performance: A Systematic Review

**DOI:** 10.1007/s40279-024-02172-4

**Published:** 2025-01-22

**Authors:** Jesús J. Ruiz-Navarro, Catarina C. Santos, Dennis-Peter Born, Óscar López-Belmonte, Francisco Cuenca-Fernández, Ross H Sanders, Raúl Arellano

**Affiliations:** 1 Aquatics Lab, Department of Physical Education and Sports, Faculty of Sport Sciences, University of Granada, Granada, Spain; 2 Department of Sport Sciences, Higher Institute of Educational Sciences of the Douro (ISCE-Douro), Penafiel, Portugal; 3 Higher Education School, Polytechnic of Coimbra, Coimbra, Portugal; 4 Section for High-Performance Sports, Swiss Swimming Federation, Bern, Switzerland; 5 Department for Elite Sport, Swiss Federal Institute of Sport Magglingen, Magglingen, Switzerland; 6 Faculty of Science and Medicine, University of Fribourg, Fribourg, Switzerland; 7 Department of Sports and Computer Sciences, Universidad Pablo de Olavide, Seville, Spain; 8 Faculty of Medicine and Health, The University of Sydney, Sydney, Australia

## Abstract

**Background:**

Swimming performance depends on a wide variety of factors; however, the interaction between these factors and their importance varies between events. In sprint events, the characterized pacing underlines its specific development, as swimmers must achieve the highest possible speed while sustaining it to the greatest extent possible.

**Objectives:**

The aim of this review was to identify the key factors underlying sprint swimming performance and to provide in-depth and practical evidence-based information to optimize performance.

**Methods:**

The review protocol was not registered. PubMed, Web of Science and Scopus databases were searched up to October 31, 2023. Studies involving competitive swimmers and investigating sprint swimming performance were included, while studies conducted with young or masters’ swimmers, triathletes or waterpolo players or not investigating sprint swimming performance were excluded. The Downs and Black Quality Assessment Checklist was performed on the included articles to assess the methodological quality.

**Results:**

After applying the PICOS framework, 39 of the 1330 articles initially identified were included according to the PRISMA guidelines. The included records focused mainly on dry-land strength and in-water forces of both upper and lower limbs. A wide range of kinematic variables were also examined, together with the importance of anthropometric and various physiological parameters.

**Conclusion:**

This review highlights the importance of developing muscular strength and effectively transferring it to performance in the water. The evidence suggests that muscular development should prioritize enhancing velocity and effective displacement, rather than merely increasing force and performance in loaded tests. However, further research is needed to confirm this. While in-water forces have been well studied, there is a notable lack of analysis regarding drag. The optimal balance between stroke rate and stroke length should be determined individually, with a primary focus on achieving a high stroke length from a high stroke rate. Although anthropometry may play an important role in performance, the interaction of these traits appears to be complex, suggesting that other factors may be more important in determining performance outcomes. From a physiological perspective, the results indicate that the lactate peak and rate of accumulation should be thoroughly developed. Notwithstanding, this review shows the lack of a solid body of knowledge on the importance of anaerobic and especially aerobic factors. Finally, the absence of a list of potential confounders, together with the lack of high-quality studies involving elite swimmers (level 1 and 2), complicates the interpretation of the results.

**Supplementary Information:**

The online version contains supplementary material available at 10.1007/s40279-024-02172-4.

## Key Points

**Table:** 

Research emphasizes the importance of developing muscular strength in the upper and lower limbs, which appears to be velocity-oriented rather than load-oriented to enhance swimming performance, although further research is needed to confirm this.
Stroke length and stroke rate play a crucial role in the development of better performance and need to be optimally combined, together with other stroke-specific factors, but the intrinsic changes that occur during their modification remain unknown.
The review highlights the need for more comprehensive studies that include elite swimmers in all four swimming strokes, as well as the lack of a thorough understanding of relevant physiological factors.

## Introduction

The goal in competitive swimming is to cover a given distance in the shortest possible time. Swimming events range from 50 to 1500 m, lasting from ~ 20 s to ~ 15 min being classified as sprint (50–100 m), middle (200–400 m), and long distance (800–1500 m) events [1]. Despite the evident difference in effort times, performance in each one of these events depends on biomechanical, physiological, and anthropometric factors [2]. Although these factors may be common across distances, their interaction and importance vary between the events [1, 3]. Therefore, these factors need to be addressed independently for each event considering the specific energetic requirements [4]. Sprint events, for instance, are characterized by an all-out or positive pacing [1]; hence, swimmers must achieve the highest possible speed while also sustaining it to the greatest extent possible [4].

To enhance swim speed, swimmers must increase propulsive forces and/or decrease drag forces [5, 6], with both dependent on a wide range of factors [2]. The complex interplay between these factors renders it exceedingly challenging to develop effective training programs [7], especially for sprinters. Swimming is predominantly considered an aerobic-based sport and, consequently, swimming coaches commonly prescribe high volumes of low-intensity aerobic training [5, 8]. Nowadays, despite a shift towards lower volume training at the highest performance levels, most training programs are still predominantly based on aerobic work [9]. While this aerobic work is indeed necessary to tolerate other types of training and enhance recovery capacity, it does not satisfy the energetic demands during actual races [10]. Thus, this circumstance has led to a discussion on whether sprint swimmers should be trained in a completely different way to match the energy systems, technical skills, and motor abilities relevant to the events. This specificity is exemplified in training modalities where intensities closely mirror an athlete's best competitive performance velocity (e.g., intermittent sprint workouts) and specific stroke aspects are emphasized during repetitions, aligning with skill acquisition principles and deliberate practice for optimal athlete development [11]. In this sense, a large amount of research in sprint swimming has emerged with the aim of understanding the key factors in performance.

To overcome the water resistance in short race distances, research has particularly focused on the effect of force production and strength on speed development [12]. These studies emphasize muscular strength, with special attention on the choice of exercises that are associated with in-water performance development [13, 14]. However, the impact of dry-land strength training on performance depends not only on the exercises used but also on the type of training and the adaptations produced. For optimal transfer to sprint performance, low-volume with high-force or high-velocity resistance training programs are recommended [15]. Yet, the specific adaptations from these training types differ [16–18], requiring careful consideration of the evaluated metrics.

Technological advancements have enabled the development of different methodologies and parameters to evaluate force in the water and these offer a wide range of possibilities with varying feasibility [19, 20]. The force application is intrinsically related to the movement, as propulsion depends not only on the force itself but also on the ability to apply this force effectively [21, 22]. Thus, kinematics plays an essential role in sprint performance. Since swimmers move at considerably higher stroke rates in sprints compared with other distances, special attention needs to be paid to the stroke mechanics. Otherwise, inefficient movements can result in energy wastage and a loss of propulsion [23–25].

Despite the short duration of the effort, sprint swimmers must maximize the energy gained [10, 26]. From the physiological perspective, given that swimming is considered an aerobic-based sport, research has primarily focused on middle- and long-distance events. However, the importance of physiology in short distances is still crucial [27, 28] and needs to be reviewed to better understand its determinants. Finally, all these factors are influenced by anthropometric characteristics, considered determinants of sprint performance [2, 29]. While somatic attributes are largely inherited, some can be modified, impacting sprint performance. Because of that, the anthropomorphological characteristics of swimmers have played an important role in the recent swimming literature that needs to be reviewed.

As comprehension of the factors in sprint would lead to better development and optimization of performance, it is necessary to provide an up-to-date review of the factors relating to sprint performance. Therefore, the aims of this systematic review were (i) to identify the dry-land strength, biomechanical, anthropometric and/or physiological factors that have been identified in the literature as influencing sprint swimming performance and (ii) to provide in-depth and practical evidence-based information to optimize sprint swimming performance.

## Methods

This systematic review was completed in accordance with the guidelines provided in the Preferred Reporting Items for Systematic Review and Meta-Analyses (PRISMA) statement [30]. The review was not registered nor was the protocol prepared beyond what is presented in this methods section.

### Search Strategy

A comprehensive and extensive search of original articles was performed encompassing publications up to October 31, 2023 in three international electronic databases: PubMed, Web of Science, and Scopus. The complete search strategy with the Boolean search method (including AND/OR) used in PubMed was as follows: ((sprint) AND (swimming)) AND ((((((((((kinematics) OR (anthropometric)) OR (strength)) OR (biomechanics)) OR (physiology)) OR (race)) OR (lactate)) OR (training)) OR (propulsion)) OR (drag)) AND (performance). Moreover, the specific search terms were modified to adjust to the nuances or requirements of the other databases as specified in Supplementary Table S1 (see electronic supplementary material [ESM]).

### Eligibility Criteria

The Population, Intervention, Comparison, Outcomes and Study (PICOS) framework [30], together with the inclusion and exclusion criteria, are described in Table 1 [31]. Furthermore, reviews (of any kind), case studies, posters, conference abstracts, or presentations were not included to ensure peer review. Studies not written in English were also excluded.

**Table 1:** Inclusion and exclusion criteria based on the PICOS framework

Item	Inclusion criteria	Exclusion criteria
Population	Healthy competitive swimmers Juvenile A or older (≥ 14.9 years)	Animals, disabled swimmers, young swimmers (< 14.9 years), triathletes, waterpolo players, or masters swimmers
Intervention	Sprint swimming, performance assessment	Middle or long distance events, open water events, nutrition, physiotherapy, health, warm-up or recovery, methodological studies (e.g., validation and reliability studies)
Comparison	Swimming distance (up to 100 m), sex	Swimming distance (longer than 100 m), age, sports, start, turn, strokes, genetics
Outcome	Sprint performance or related to it	Not related to sprint performance
Study design	Cross-sectional	Longitudinal (intervention)

### Study Selection

The selection of relevant articles was carried out by two independent researchers, both PhD holders with previous experience in conducting systematic reviews. First, all studies retrieved from the databases were screened, duplicate articles were removed, and titles and abstracts were inspected independently. The eligibility criteria (Table 1) were applied by both researchers and disagreements were discussed until a consensus was reached. The same procedure was then followed after the full-text screening of the remaining articles for the final decision. Finally, the reference lists of the included articles were reviewed to identify articles that might not have been found in the initial search. However, no further articles were identified for inclusion.

### Data Extraction

The extraction process was conducted by one researcher and double-checked by another independent researcher. The items extracted were (i) study reference; (ii) main purpose; (iii) number of participants per sex, age, and competitive level; (iv) assessment protocol; and (v) main findings.

### Quality Assessment

Two independent reviewers performed the quality assessment of each study. In case of disagreements and uncertainty, a third reviewer was consulted. The Downs and Black Quality Assessment Checklist [32] was used based on the following criteria: reporting, external validity, internal validity (bias and confounding), and power. This tool has been employed in systematic reviews within the sports domain [19, 33, 34].

In alignment with the study focus and previously adapted versions, the following adjustments were made [19, 33, 35]: replacing ‘patient’ with ‘participant’ and ‘treatment’ with ‘testing’; items not applicable to the study design i.e., cross-sectional study were excluded (4, 8, 9, 14, 15, 17, 19 and 22–26); and the response format for item 27 was simplified to ‘yes’ (1 point) or ‘no’ (0 points), rather than offering five options (Supplementary Table S2 in the ESM). Methodological quality was categorized as low (≤ 50%), good (51–75%), or excellent (> 75%) [36] with the percentages calculated as (manuscript score / 16 (maximum score)) × 100.

Inter-rater reliability, reflecting the degree of agreement between reviewers during the scoring process, was assessed using Cohen’s Kappa coefficient (*κ*) [37]. Interpretation followed Landis and Koch’s suggestion [38]: no agreement if *κ* < 0; poor agreement if 0 < *κ* < 0.19; fair agreement if 0.20 < *κ* < 0.39; moderate agreement if 0.40 < *κ* < 0.59; substantial agreement if 0.60 < *κ* < 0.79; and almost perfect agreement if 0.80 < *κ* < 1.00.

## Results

### Article Identification

**Fig. 1:**
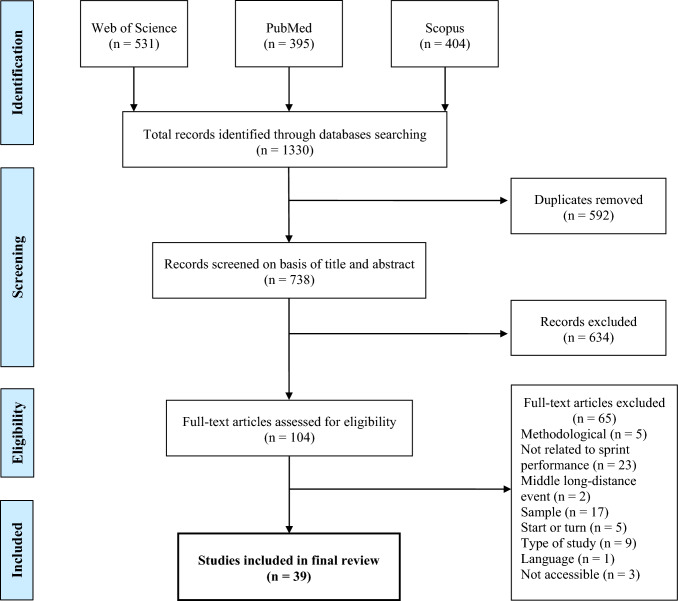
Preferred Reporting Items for Systematic Reviews and Meta-Analyses (PRISMA) flowchart of the study selection process

The initial search identified 1330 records. After duplicate removal, 738 records were manually screened by title and abstract, which resulted in the exclusion of 634 records. The full texts of 104 records were assessed for eligibility and 65 of those were excluded. For instance, the study by Gatta et al. (2012) [39] was potentially considered as it provided valuable information about flutter kick propulsion; however, this was not integrated in whole body propulsion. Also excluded was the study by Flatt et al. (2017) [40], which examined changes in heart rate variability and wellness parameters in response to different training periods but did not assess their impact on performance. Hence, a total of 39 articles were considered for further analysis. The complete and detailed search process is shown in Fig. 1.

### Quality of Included Studies

The inter-rater reliability analysis showed an almost perfect agreement (*κ* = 0.83) among raters in the scoring process using the quality index. A comprehensive summary of the quality index for each study is presented (%) in Table 2 while the individual quality index outcomes are presented in Supplementary Table S3 (see ESM). The overall quality index exhibited a mean (± standard deviation) percentage score of 55.1 ± 9.7% (ranging from 37.5% to 75%). Notably, several studies lacked a list of potential confounders and reporting statistical power. On the other hand, the studies consistently presented clear descriptions of the main outcomes to be measured, stated their main findings, and provided estimates of random variability.

**Table 2:** Summary of the main purpose, participants’ background, assessment protocol and main findings reported by the studies included in this review. The table is structured by main outcome domain (please note that some articles cover different domains), within which studies are ordered from high to low performance level

Study reference	Quality index (%)	Main purpose	Sample (sex, age, level)	Assessment protocol	Main findings
Dry-land strength					
Nicol et al. [74]	56.25	To evaluate the relationship between dry-land strength and power, and BR kinematics	♂ *n* = 5 ♀ *n* = 6 23.0 ± 3.0 y (pooled) Level 1	Anthropometric Passive ROM 3 × CMJ 3 × Pull-up 3 × 3-s max. isometric adductor test 3 × 25-m BR (100 and 200 m, maximal paces)	Update profile of ROM, strength-power and anthropometric characteristics of level 1 breaststrokers was provided Both sexes showed several strong relationships between dry-land strength and breaststroke kinematics
Carvalho et al. [72]	56.25	To investigate the linear relationships between sprint performance in the four strokes and upper and lower limb strength	*n* = 16 ♂ *n* = 9 ♀ *n* = 7 20.7 ± 3.3 y Level 2 *n* = 14 ♂ *n* = 8 ♀ *n* = 6 15.9 ± 1.7 y Level 4	Anthropometric 4 × 25-m BU, BA, BR, FC max. effort 10 × maximal isokinetic contraction at 90 and 300º/s 3 × CMJ	Upper and lower limb strength were moderately to largely correlated with sprint performance in the four swimming strokes BU and FC sprint performance primarily depended on SL BA and BR sprint performance primarily depended on SR Arm spam was the strongest anthropometric predictor of BU, BA, and FC
Keiner et al. [13]	50	To examine the relationships between strength, jump performance, and swimming performance, including start and turn performances	♂ *n* = 14 17.5 ± 1.6 y Level 3	18-m semi-tethered FC (1.33 kg) 2 × 50-m FC max. effort 2 × 100-m FC max. effort 5–8 × CMJ 5–8 × SJ 1RM bench press test 1RM back squat test	The 50 and 100-m sprint swimming performance were predicted by 1RM in bench press and back squat Absolute 1RM in bench press and back squat values showed better association with 50-m performance relative to body weight values
Amara et al. [42]	43.75	To examine the potential relationship between the predicted 1RM push-up and FC swimming performance and kinematics	♂ *n* = 33 16.4 ± 0.6 y Level 4	4 × 3 push-ups – body weight, 10, 20, and 30-kg weight vests) 25 and 50-m FC max. effort 25 and 50-m FC arm stroke max. effort	The findings showed a nearly perfect correlation among the 1RM push-up and 25 and 50-m FC full and arm stroke swimming performances A nearly perfect association was observed between the 1RM push-up and SL as well as SR
Loturco et al. [14]	50	To determine the exercises and the mechanical variables related to tethered swimming variables	♂ *n* = 10 17.0 ± 0.7 y Level 4	50, 100, and 200-m FC max. effort 2 × 10-s tethered test 5-s isometric bench press (90º) and quarter squat (135º) tests Mean propulsive power test in jump squat and bench press 5 × CMJ and SJ	Tethered swimming demonstrated a large to very large correlation with 50 and 100-m swimming performance Lower limb power tests, conducted under both loaded and unloaded conditions, exhibited large to very large correlations with tethered forces Mean propulsive power measured during jump squats was largely associated with 50-m swimming performance
Morouço et al. [59]	50	To determine which specific dry-land tests exhibit a stronger association with tethered variables and sprint performance	♂ *n* = 10 14.9 ± 0.7 y Level 4	30-s whole-body tethered test 30-s arms-only tethered test 30-s kick-only tethered test 50-m FC max. effort Bench press, squat and lat pull-down RM test 3 × CMJ	Maximum mean power of the propulsive phase during the lat-pull down was the only parameter that correlated with swimming performance Average force in whole-body condition was correlated with all four exercises Average force in arms-only condition was correlated with maximum mean power of the propulsive phase in bench press and lat pull-down Average force in kick-only condition was correlated with maximum mean power of the propulsive phase in squat and CMJ work
Perez-Olea et al. [51]	62.5	To assess the relationship between pull-ups and the CMJ with sprint swimming performance	♂ *n* = 12 19.0 ± 3.0 y Level 4	5 × CMJ 30 × CMJ 5 × pull-ups Max. repetitions of pull-ups 50-m FC max. effort 50-m flutter kick max. effort	The mechanics (velocity and power) during the pull-up are indicative of swimming performance, whereas the total number of pull-ups an athlete can perform is not predictive The CMJ showed no correlation with flutter kicking or FC swimming performance. This underscores the notion that other technical factors, such as body position or leg-kick effectiveness, play more significant roles than lower-limb strength
Chalkiadakis et al. [67]	56.25	To examine the associations between dry-land variables derived from F–V and P–V profiles, in-water force variables, and swimming performance and kinematics in 50–400 m and 4 × 50 m	♂ *n* = 9 17.3 ± 3.6 y Level 5 ♀ *n* = 6 15.7 ± 1.9 y Level 5	Bench press 1 RM test 10-s tethered test 50, 100, and 200-m FC max. effort 4 × 50-m FC max. effort	Dry-land and in-water force variables were related to sprint performance Maximum power showed the highest association with sprint performance Tethered forces were related to SR and SI during sprint events
Özkadı et al. [68]	62.5	To examine the anthropometric and motoric parameters associated with 50-m performance in the four swimming strokes and explore sex differences	♂ *n* = 20 16.5 ± 0.5 y Level 5 ♀ *n* = 20 16.5 ± 0.5 y Level 5	Anthropometric Squat horizontal jump Handgrip test Sit-up test Sit and reach test Trunk – neck test Shoulder mobility Cooper test 30-m running speed Illinois test Flamingo test BU, BA, BR, and FC 50-m official race time	50-m swimming performance in all four strokes were associated with squat horizontal jump and aerobic performance in both sexes Agility, balance, and flexibility were also determinants of sprint swimming styles in females Abdominal muscle endurance presented a positive association with all the strokes except BU in females with the exception of FC in males The flexibility presented positive association with all the strokes except BU in males Running speed was associated with FC and BU in both sexes Body height, hand and foot lengths could be important indicators for swimming strokes
Keiner et al. [75]	37.5	To study the variables that determine the influence of maximal strength performance on swimming strokes performance and distances	♂ *n* = 12 ♀ *n* = 9 17.5 ± 2.0 y (pooled) Not reported	15, 25, 50, and 100-m FC max. effort 50 and 100-m BR max. effort 50 and 100-m BA max. effort 15 and 25-m BR arm stroke max. effort 25-m BR kick max effort Squat, bench press, sit-up, bent-over row, and deadlift 1 RM test CMJ and SJ test	Lower and upper limb strength were related to swimming performance, especially for FC and BU events Trunk strength was related to swimming performance
Kinetics					
Gatta et al. [73]	75	To analyze the association between mechanical power output, propelling efficiency and velocity	♂ *n* = 12 22.8 ± 3.5 y Level 2	15-s tethered test 15-s whole-body swimming ergometer max. effort 8 × 25-m FC even paced incremental speed	Maximal sprint swimming depended on the interplay between power output in dry conditions and propelling efficiency Power output was better estimated by means of the tethered swimming test than with a laboratory-based ergometer
Gonjo et al. [62]	56.25	To analyze the associations between sprint swimming and kinematics and L–V profile variables in BU swimming	♂ *n* = 12 19.8 ± 2.5 y Level 3	50-m BU max. effort 3 × 25-m BU semi-tethered (1, 5, and 9 kg)	Three loads were enough to properly establish the individual L–V profile in butterfly swimming Validity of the L–V profile to predict 50-m swimming performance
Gonjo et al. [43]	50	To investigate the associations between L–V profile variables and 50-m FC swimming performance	♂ *n* = 14 19.9 ± 3.2 y Level 4	Anthropometric 50-m FC max. effort 3 × 25 FC semi-tethered max. effort (1, 5, 9 kg)	Sprint swimming performance was largely to very largely correlated with the L–V parameters The anthropometrics were associated with the maximum load at zero velocity, but not with the maximum velocity at zero load
Morais et al. [3]	68.75	To establish the main determinants of FC swimming speed	♂ *n* = 10 16.4 ± 0.7 y Level 4 ♀ *n* = 13 14.9 ± 0.9 y Level 4	3 × 25-m FC max. effort 50-m FC max. effort 400-m FC max. effort 2 × 25-m FC max. effort: - 1 free - 1 towing a hydrodynamic body	FC swimming speed was a multifactorial phenomenon related to faster SR, lower active drag coefficient, higher blood lactate, and lower critical speed There was no sex effect in swimming speed models Swimming speed decreased throughout the trial
Morouço et al. [47]	50	To evaluate the magnitude and relationship of upper limb kinetic asymmetries in FC tethered swimming	♂ *n* = 18 15.6 ± 2.1 y Level 4	30-s tethered test 50-m FC max. effort	66.7% of the swimmers exhibited asymmetry in the force developed towards dominant upper limb superiority, with opposite breathing laterality Higher force asymmetry did not negatively impact swimming performance, but it did emerge as a significant factor to consider when controlling the relationships between exerted forces and performance
Morouço et al. [48]	62.5	To examine the association between 30-s tethered variables and blood lactate with FC sprint performance	♂ *n* = 7 16.6 ± 1.0 y Level 4 ♀ *n* = 6 15.8 ± 0.8 y Level 4	Anthropometric 30-s tethered test 50 and 100-m FC official race time	Mean and maximum tethered forces are related to 50 and 100-m FC performance Fatigue index was not related to 50 or 100-m FC performance Fatigue slope was correlated with 50 and 100-m FC performance and with blood lactate concentration
Morouço et al. [49]	50	To compare kinematic and physiological responses between tethered and free swimming To analyze the associations between tethered force parameters and FC swimming speed	♂ *n* = 23 17.2 ± 2.7 y Level 4	30-s tethered swimming 50-m FC max. effort	Same SR and physiological responses between tethered and free swimming of similar duration and intensity Tethered swimming might be used as a tool to assess the balance between force and the ability to effectively apply force The impulse of force rather than maximum force should be used as a determinant for explaining swimming performance during swimming at high speeds
Ruiz-Navarro et al. [52]	75	To examine variables that might be used to quantify swimmers’ ability to apply force in the water and to test their relationship with free swimming performance	♂ *n* = 16 19.6 ± 3.3 y Level 4	4 × 30-s FC arm stroke tethered test at different water flow velocities 25, 50, and 100-m FC max. effort	The relative changes in maximum and average force between arm-stroke tethered swimming at zero and 1.389 m/s water velocity could be used to quantify the ability of swimmers to exert force in the water regardless of muscle strength The proposed parameters were strongly associated with sprint swimming performance
Schreven et al. [54]	56.25	To evaluate and compare the impact of power, technique, and anthropometric measures on sprint performance during arms-only FC swimming	♂ *n* = 25 22.0 ± 5.0 y Level 4	Anthropometric 4 × 25-m FC arm stroke max. effort MAD measurement 10–12 × 23-m FC arm stroke progressing speed	Power-to-drag ratio was the only predictor of swimming speed Variations in the maximal power-to-drag ratio explained 65% of the variance in the swimming performance
Ruiz-Navarro et al. [53]	43.75	To investigate the correlations between two swim-specific measures of anaerobic performance and dry-land strength-based variables To explore the associations among the identified variables and swimming performance and kinematics To explore the potential sex-induced differences	♂ *n* = 14 17.4 ± 2.9 y Level 5 ♀ *n* = 9 17.3 ± 2.4 y Level 4	5 × CMJ 5 × pull-ups 50-m FC max. effort 30-s tethered swimming 10, 15, 20 and 25-m FC max. effort (anaerobic critical velocity test)	Sprint swimming performance was associated with anaerobic critical velocity, tethered forces, CMJ, and pull-ups in both sexes There is a sex-induced difference when comparing males and females, as males relied more on upper body and females on lower body strength
Silva et al. [70]	56.25	To identify the crucial variables for analyzing the impact of both sex and skill on sprint performance	♂ *n* = 23 15.7 ± 0.8 y Level 5 ♀ *n* = 26 14.5 ± 0.8 y Level 5	Anthropometric Shoulder mobility 50-m FC max. effort 30-s tethered swimming 25-m active drag measurement	The main difference between swimmers’ levels was associated with swimming efficiency, being determinant of males’ performance There is a clear difference in anthropometrics, performance, and kinematics between sexes at the end of the maturational process
Rozi et al. [76]	56.25	To assess performance in 100-m FC using an equal-duration tethered swimming test	Sex not defined *n* = 23 15.0 ± 1.6 y Not reported	Anthropometric 100-m FC max. effort Tethered swimming test of duration equal to 100-m time Handgrip test	Swimming speed performance was highly associated with tethered forces, handgrip, and biceps circumference
Kinematics					
Barbosa et al. [78]	56.25	To examine the correlation between 50-m FC performance and speed curve variables To analyze and identify stroke cycle differences in speed curves of 23, 22, and 21-s swimmers	♂ *n* = 14 25.7 ± 6.4 y Level 2	50-m FC official race time 25-m FC max. effort	Sprint performance showed very large correlation with mean and peak speed Sprint performance did not show association with minimum speed or intracyclic velocity variation Faster swimmers were able to reach higher speeds and prolong their duration within the upper part of the speed curve
Gourgoulis et al. [63]	50	To examine the leg kick influence on hand kinematics and propulsion and overall swimming kinematics	♀ *n* = 9 18.4 ± 4.9 y Level 3	2 × 25-m FC max. effort: 1 arm stroke 1 full stroke	Kicking evoked higher swimming speed, SL, and SR Kicking did not affect stroke kinematics and kinetics Kicking caused a decrease in trunk inclination
Simbaña-Escobar et al. [65]	62.5	To investigate the influence of sex and manipulated SRs on FC swimming performance and arm coordination To examine how the preferred SR may affect the adaptation of swimmer behavior	♂ *n* = 11 20.7 ± 3.2 y Level 3 ♀ *n* = 8 21.3 ± 3.7 y Level 3	2 × (9 × 25 m) FC max. effort at different SR: Preferred, maximum, 41, 44, 47, 50, 53, 56, 59 cycles/min	Higher SR led to an increase in swimming speed The maximal SR was higher than the preferred SR The increase in SR led to a higher index of coordination The changes in speed and index of coordination did not occur independently of the preferred SR. The further away from the preferred SR, the higher the error Female swimmers struggled to sustain the prescribed SR above their preferred SR, while males exhibited a broader range of SRs including a higher preferred stoke rate than females
Takeda et al. [66]	43.75	To assess the persistence of initial speed differences throughout the stroke phase in FC swimming – kinematics	♂ *n* = 10 20.1 ± 1.0 y Level 3	3 × 25-m FC max. effort (max. effort dive, submaximal-effort dive, max. effort wall push)	The initial speed is highest during the race Swimming speed was the same during the strokes regardless of differences in initial speed
McCabe et al. [44]	50	To examine the impact of breathing on ipsilateral upper limb kinematics during FC sprint swimming compared with non-breathing strokes and evaluate its influence on performance	♂ *n* = 10 18.4 ± 2.6 y Level 4	25-m FC max. effort no breathing 25-m FC max. effort breathing throughout the trial	Lower swimming speed when breathing, with a negative tendency in both SR and SL During the entry phase, swimmers exhibited a reduced horizontal velocity, along with decreased shoulder flexion, abduction, and roll in the breathing trial The pull phase extended in duration, presenting a shallower hand path, diminished shoulder abduction, slower hand vertical acceleration, and reduced velocity when breathing The push phase showed a shortened duration, with swimmers decreasing the range of elbow extension, faster hand horizontal velocity, and greater hand vertical acceleration when breathing
Morais et al. [60]	68.75	To predict swimming velocity using a set of anthropometric kinematic and kinetic variables To investigate the SR–SL combinations linked to swimming velocity and propulsion	♂ *n* = 25 15.9 ± 0.7 y Level 4 ♀ *n* = 9 14.9 ± 1.0 y Level 4	Anthropometric 3 × 25-m FC max. effort	Males were faster than females Anthropometric features exert a positive and significant impact on swimming velocity only when coupled with increased muscle strength Swimming speed was predicted by the height, underwater stroke time, and mean force The highest speed was not achieved at the highest SR or SL The highest propulsion was not responsible for producing the fastest swimming velocity
Morais et al. [46]	68.75	To examine whether a hypothetical variation in determinant factors between the upper limbs may be associated with maximum FC speed To identify the primary predictors influencing swim speed during each upper-limb arm-pull – kinematic	♂ *n* = 22 15.9 ± 0.7 y Level 4	Anthropometric 3 × handgrip trials 3 × 25-m FC max. effort	Swimmers exhibited significant disparities in upper limb anthropometrics, thrust, and speed, while dry-land strength showed non-significant differences Swimmers struggled to sustain their thrust and speed in both upper limbs during the trial The speed achieved by each upper limb was influenced by a complex interplay of factors, particularly thrust and kinematics
Strzała et al. [55]	37.5	To evaluate the impact of somatic features and anaerobic power on sprint surface BU swimming – kinematics	♂ *n* = 34 19.3 ± 1.8 y Level 4	Anthropometric 3 × CMJ 50-m BU max. effort	Butterfly sprint performance was associated with SR, spatial–temporal indices (entry-kick, fly-arm, first kick) A more nuanced understanding of the matter can be attained by exploring the inter-correlations among the temporal indices
Strzała et al. [57]	43.75	To quantitatively assess stroke kinematics and coordination in sprint BR – kinematics To characterize trunk behavior in relation to swimming speed To investigate the inter-relationships between the indicators of stroke kinematics, swimmers’ sacrum accelerations and pitch rotation	♂ *n* = 34 19.1 ± 1.9 y Level 4	Anthropometric 50-m BR max. effort	Breaststroke sprint performance was highly associated with SR and arm total propulsion phase duration The acceleration of the sacrum in the ventral direction during arm recovery appears to be connected to wave action acceleration, contributing to enhanced swimming velocities
Anthropometrics					
Dopsaj et al. [61]	50	To establish the correlations between swimming performance and body composition characteristics To define a multidimensional model of performance prediction	♂ *n* = 46 22.9 ± 4.2 y Level 3 ♀ *n* = 36 21.0 ± 4.7 y Level 3	Body composition BU, BA, BR, or FC 50 and 100-m official race time	Male swimmers’ performance is associated with a balanced ratio of contractile and non-contractile tissue, along with a high level of muscle tissue Female swimmers’ performance is associated with a high level of muscle tissue and a proper low level of fat The defined body composition models explained 35.1% and 75.1% of the mutual variability in performance for males and females, respectively, with standard errors of 57 WA points
Strzała et al. [56]	56.25	To examine the relationship between 100-m performance and in-water and dry-land variables	♂ *n* = 26 19.8 ± 2.4 y Level 4	Body composition 40-s arm-crank max. effort 20 × CMJ 40-s arm stroke tethered swimming at 0.9 m/s water flow velocity 40-s tethered flutter kick 100-m FC max. effort	Fat-free mass and total body water were associated with swimming speed only when assessed using absolute values, not when considered relative to body weight Fat-free mass showed association with absolute limb strength in dry-land conditions, but no correlations were observed when normalized to body weight
Siders et al. [77]	50	To identify the correlations between body composition, somatotype components, and sprint swimming performance	♂ *n* = 31 20.5 ± 1.9 y Not reported ♀ *n* = 43 19.7 ± 1.4 y Not reported	Anthropometric BU, BA, BR, or FC 100-yard competitive event	Female performance was positively associated with body height, fat-free mass and ectomorphic somatotype and negatively associated with mesomorphic somatotype Male performance was not associated with anthropometric characteristics
Physiological factors					
Mavroudi et al. [64]	50	To analyze the blood lactate response to maximal sprint efforts	♂ *n* = 8 21.7 ± 4.8 y Level 3 ♀ *n* = 6 18.2 ± 3.0 y Level 3	25, 35, and 50-m max. effort at the specialized stroke (BU, BA, BR, or FC)	VLa_max_ was higher as the swimming distance decreased VLa_max_ was correlated to swimming speed in every distance The time to reach the peak blood lactate after the exercise did not differ between swimming distances
Merati et al. [45]	62.5	To analyze the relationship between autonomic modulations of HR and sprint performance	♂ *n* = 13 22–32 y Level 4	Heart rate variability measurement (baseline, before training, after training) 50 and 100-m FC official race time	The HR vagal modulation exhibited a positive correlation with 50-m time Cardiac sympatho/vagal balance, measured post-training, showed a negative correlation with 100-m time
Noriega-Sánchez et al. [50]	75	To examine the association between anthropometric, conditioning, and pulmonary function variables on 100-m FC performance	♂ *n* = 8 19.4 ± 0.7 y Level 4 ♀ *n* = 9 16.9 ± 3.2 y Level 4	Anthropometric 100-m FC max. effort 3 × 1 maximal inspiration and enforced exhalation 3 × SJ 3 × CMJ	Forced inspiratory volume in the first second explained 66% and 58% of the 100-m FC performance variance in males and females, respectively Significant differences in pulmonary function, anthropometric, and conditional parameters among sexes
Terzi et al. [58]	56.25	To investigate the relevance of a maximal 4 × 50-m FC training set in 100-m FC performance, blood lactate and kinematics	♂ *n* = 11 16.0 ± 1.3 y Level 4 ♀ *n* = 16 16.2 ± 1.0 y Level 4	4 × 50-m FC max. effort 100-m FC max. effort	Swimming speed, lactate, and SR were higher in the 4 × 50-m set than in 100-m FC Speed, lactate, SR, and SI were associated among tests The 4 × 50-m set is a suitable and adequate training stimulus for improving 100-m performance
Rodriguez et al. [69]	43.75	To explore the VO^2^ kinetics during maximal FC whole stroke 100-m swimming and arm stroke and leg kick exercises of equal duration	♂ *n* = 26 15.5 ± 2.2 y Level 5 ♀ *n* = 10 15.4 ± 1.8 y Level 5	100-m whole-body FC max. effort 100-m arms-only FC max. effort 100-m kick-only FC max. effort	Lower oxygen uptake amplitude is reached with arms-only and kick-only compared with whole-body swimming Whole-body elicited a similar rate of oxygen uptake to kick-only and a higher rate than arms-only 100-m performance was associated positively with the oxygen uptake amplitude and inversely with the time delay of the fast component

♂ males, ♀ females, *BA* backstroke, *BR* breaststroke, *BU* butterfly, *CMJ* countermovement jump, *FC* front crawl, *F–V* force–velocity, *HR* heart rate, *HRV* heart rate variability, *L–V* load–velocity, *P–V* power-velocity, *RM* repetition maximum, *ROM* range of motion, *SI* stroke index, *SJ* squat jump, *SL* stroke length, *SR* stroke rate, *VLa*_*max*_ maximal blood lactate accumulation rate, *WA* World Aquatics points, *y* years

### Description of the Included Articles

The characteristics of the records included are presented in Table 2, which has been structured by the main outcome domain (please note that some articles cover different domains), within which studies are ordered from high to low performance level to facilitate the results comparison from different studies. There were no eligible records prior to 1993. Most of the eligible records (35 of 39) were published between 2013 and 2023. The study populations, following the proposed classification model [41], were as follows: 53.8% Level 4 (21/39) [3, 14, 42–60], 17.9% Level 3 (7/39) [13, 61–66], 10.2% Level 5 (4/39) [67–70], 7.6% Level 2 (3/39) [71–73], and 2.5% Level 1 (1/39) [74]; 7.7% did not report the swimmers’ performance level (3/39) [75–77]. Please note that two of the manuscripts [53, 72] reported samples with two different levels and only the highest level has been used to provide the percentages. Regarding sex, 17 of the records had both male and female participants [3, 48, 50, 53, 58, 60, 61, 64, 65, 67–70, 72, 74, 75, 77], 20 had all male participants [13, 14, 42–47, 49, 51, 52, 54–57, 59, 62, 66, 73, 78], one had all females [63], and the participants’ sex in the remaining study was not reported [76]. The sample mean age ranged from 16 to 25 years, with 18 records having swimmers with a mean age under 18 years [3, 13, 14, 42, 46–49, 53, 58–60, 67–70, 75, 76] (two of them had both under and over 18 years) [50, 72].

The most studied stroke was front crawl, being explored in 35 of the studies [3, 13, 14, 42–54, 56, 58–61, 63–73, 75–77]. Butterfly [55, 61, 62, 64, 68, 72, 75, 77] and breaststroke [57, 61, 64, 68, 72, 74, 75, 77] were analyzed in eight studies, and backstroke in only six of the records [61, 64, 68, 72, 75, 77]. Full-stroke swimming was analyzed in 38 of the studies [3, 13, 14, 42–53, 55–77], arm-only swimming in eight [42, 52, 54, 59, 63, 69, 75], and leg kick in five of them [51, 56, 59, 69, 75].

Five subsections of studies were identified. (i) Dry-land strength: of the 39 studies included in the review, 16 explored the relationship between resistance exercises (i.e., body weight or non-body weight exercise) and sprint performance or kinematics [13, 14, 42, 46, 50, 51, 53, 55, 56, 59, 67, 68, 72, 74–76]. Five of the aforementioned studies analyzed body weight exercises [50, 51, 53, 55, 56], seven focused on both body weight and non-body weight exercises [13, 14, 42, 59, 68, 72, 74, 75], and four explored only non-body weight exercises [42, 46, 67, 76]. (ii) Kinetics: the relationship between tethered parameters and swimming performance was explored in 12 studies [14, 47–49, 52, 53, 56, 59, 67, 70, 73, 76], while semi-tethered was analyzed in three manuscripts [13, 43, 62]. Active drag was measured in three studies [3, 54, 70]. (iii) Kinematics: a wide range of kinematic factors were explored in 18 of the records included in this review [3, 42, 44, 46, 49, 53, 55, 57, 58, 60, 63, 65–67, 70–72, 74]. (iv) Anthropometrics: the anthropometrics was an object of study in 11 articles [46, 50, 55, 56, 60, 61, 68, 70, 72, 74, 77]. (v) Physiological factors: physiological measurements were taken in eight studies [3, 45, 49, 50, 58, 64, 68, 69].

## Discussion

This systematic review aimed to identify the neuromuscular, biomechanical, anthropometric, and/or physiological factors that have been identified in the literature as influencing sprint swimming performance and to provide in-depth and practical evidenced-based information to optimize sprint swimming performance. A considerable amount of research has been conducted to address the importance of dry-land strength, kinetics, kinematics, and anthropometrics. However, other factors such as active drag or physiological measurements require further research. Overall, the included studies demonstrated good methodological quality. However, the quality ranged from low to good, with none reaching an excellent standard.

### Dry-Land Strength

In swimming, most of the applied force (of the upper body) stems from the back muscles [79], and as such, arm pull tests are crucial for evaluating upper body strength and endurance. Research indicates that the pull-up and lat pull-down exercises are positively associated with performance across various factors [51, 53, 59, 74]. These exercises target the latissimus dorsi, a key muscle involved in the vertical plane of motion [5]. It is therefore not surprising that the aforementioned are two of the most prescribed exercises by elite strength and conditioning coaches in swimming [80, 81]. Nevertheless, among the different parameters that can be measured from these exercises, research shows that the velocity and power developed during the concentric phase of a single arm pull-up and lat pull-down or a maximum number of repetitions test show the strongest associations with performance [51, 53, 59, 74], whereas other factors such as the total number of pull-ups do not show any association [51]. This discrepancy may be attributed to neuromuscular differences between force and speed production and their respective maintenance over time. In light of these findings, it appears essential for swimmers to prioritize rapid force production, regardless of the actual movement speed, to evoke the greatest improvements in swimming speed [82]. The results suggest that the benefits derived from dry-land strength training are not solely dependent on the exercise selected but more importantly on the characteristics of the movement execution, specifically the maximal intended velocity.

The repetition maximum of a weighted push-up as well as bench press, both exercises that stimulate the pectoralis major which is highly involved in propulsion [83], were positively associated with front crawl swimming performance [13, 42, 67]. The association is stronger when absolute values are used rather than when values are relativized to body mass [13]. The reason for this higher association could be attributed to the effect of buoyancy (a force influenced by the body's specific mass and density), which counteracts body weight in the water and, consequently, its effect on swimming [84]. It is important to note that in the scientific literature, the bench press is likely the most studied upper-body exercise in terms of force/load-velocity profile [85, 86]. However, there is only one study specific to sprint swimming [67]. The results showed that the velocity of the movement evidenced a slightly better association with swimming performance than force production. Hence, in line with the pull-up results [51, 53, 74], swimmers might need a more oriented velocity profile. Notwithstanding, the literature remains scarce regarding the study of load/force–velocity profile in dry-land exercises for swimming. Thus, based on findings from other disciplines [87], swimming research should aim to explore in-depth load/force–velocity profiles to better orientate dry-land strength training.

To a lesser extent, the studies included in this systematic review also examined isokinetic and isometric exercises [14, 72, 76]. The findings indicate, except for handgrip, which is an indicator of overall strength [88], that the force generated at zero velocity (i.e., isometric) is not associated with performance, whereas the force developed at various speeds, particularly at very high speeds, shows a positive relationship with performance. Overall, these findings are in line with previous results, underscoring the importance of the velocity as swimmers need to apply a higher amount of force at relative high velocities, especially considering that the underwater hand path should be performed with progressively increasing speed [89]. Hence, the highest propulsion should be achieved at the end of the stroke when the hand speed is the highest [5, 90]. Yet, the low number of studies investigating this type of exercise means further research is required.

Core muscle development is one of the main goals of elite swimming strength and conditioning coaches during dry-land training [80, 81]. In this regard, the two included studies examining core strength found positive associations with swimming performance using a maximal and an endurance sit-up test [68, 75]. This phenomenon relies on the basis that a stronger core is crucial to overcome the unstable and dynamic nature of the water [91], as well as to ensure the transference of force between the upper and lower limbs, hence granting an efficient locomotion [91]. Moreover, despite the non-specificity of the exercise, this result might indicate that the core plays a role in propulsion beyond the transfer of force. Indeed, torso twist was highlighted as a supplementary function by the torso muscles [23]. Therefore, designing exercises that challenge the torso muscles to generate torques that produce or resist longitudinal rotation of the upper and lower torso could transfer to improvements in swimming performance [92].

When exploring the relationship between sprint swimming performance and lower limb strength, mixed findings are shown. Several studies evidenced association for all four strokes in a wide variety of factors (jump height, work, or flight time) and exercises (countermovement jump, squat jump, squat horizontal jump, squat, loaded squat jump) [13, 14, 53, 68, 72, 74, 75], while a large number of other studies showed a lack of such correlations [50, 51, 55, 56, 59]. The potential explanation for these contradictory results may lie in the propulsive role of the leg kick. Despite swimming speed increasing when kicking [63], the propulsion generated by the lower limbs (except in breaststroke) is considerably lower than the propulsion contributed by the upper limbs [63, 93–95]. Hence, swimmers that highly rely on upper limb propulsion may benefit less with little benefit of improved leg strength on swimming velocity. Furthermore, when kicking, no changes are evoked on the hand’s kinematics but a decline in drag is observed due to the reduction in trunk inclination [63], which indicates that other technical factors such as body position, leg-kicking technique, and ankle flexibility may play more important roles than lower-limb strength [96]. Interestingly, the association between leg strength and kicking performance was stronger in swimmers with higher performance level [53, 72]. Swimmers with higher performance level, and hence higher technical skills, may have a larger benefit of muscle power in the lower limbs as a fundamental aspect of enhancing sprint performance. Hence, these findings support the development of kicking and intensive effort put into leg series during training as this higher propulsion and drag reduction seems crucial in the pursuit of success; however, future research should elucidate the impact of lower limb strength across populations with varying performance and skill levels.

### Kinetics

Tethered swimming is a reliable method for measuring mechanical outputs in aquatic environments, being extensively recognized as a powerful tool to assess the specific forces applied by swimmers during specific movements [14, 73]. As such, tethered swimming showed a close association with sprint performance in a large number of studies using different parameters [14, 47–49, 52, 53, 56, 59, 67, 70, 73, 76]. From a mechanical point of view, it is expected that swimmers capable of applying higher amounts of force/power against the water will achieve higher swimming speed [97]. However, the force applied not only depends on the swimmers’ muscular force production [14, 53, 56, 59, 98] but also on their ability to apply that force [12, 21, 52]. Hence, swimming kinematics has an impact on the propulsion generated [2]. For instance, the peak force is typically achieved at a single point within the arm stroke cycle. While this point at which the peak force occurs is crucial for propulsion, swimmers increase their hand speed throughout the underwater path [89, 90], which results in a more continuous force production. In contrast to on-land sports such as running that aim for maximal force production within minimal ground contact time, swimmers that sustain lower force levels throughout longer arm strokes can yield comparable, if not greater, momentum changes than those resulting from higher forces applied over shorter durations [99]. Since the impulse takes both force and time of application into account, it seems that the impulse of force should be considered, especially with higher-level swimmers, as they may take advantage from every part of the underwater path [21, 49].

Technological advancements have facilitated the measurement of force while swimming. As such, semi-tethered swimming allows the swimmers to move forward while displacing an external load [100, 101]. This approach appears to overcome the missing specificity of force production during tethered swimming (due to the fixed position), measuring the velocity with different external loads to generate load-velocity profiles [62, 101]. From the load-velocity profile, both V_0_ (the maximum velocity at zero load) and L_0_ (maximum load at zero velocity) showed positive association with swimming performance in butterfly and front crawl [43, 62]. However, the association was indeed lower in L_0_ than in V_0_. This implies that swimmers need to apply a large force to the water, but this force needs to be effectively applied to produce high speed [43, 52]. In this sense, monitoring of these two parameters would likely indicate whether swimmers have maximized propulsion or minimized resistance [43].

It is important to note that the highest speed during the stroke is not achieved at the highest propulsion, as the latter may occur under conditions of elevated drag, thereby resulting in diminished velocity [60]. Despite the importance of drag, its impact during sprint swimming has been explored to a lesser extent than propulsion [3, 54, 70]. No direct association between drag and performance has been observed. However, drag is often included in more complex predictive models, which underscores its importance [3, 54]. This can be explained by the fact that displacement through the water depends on both propulsion and drag. Therefore, low levels of drag per se cannot produce high speeds unless accompanied by a certain level of propulsion. Indeed, the power to drag ratio showed a better association with swimming speed than propulsion or drag alone [54]. This result suggests that any training intervention aiming to increase propulsion must therefore be conducted with consideration of its effects on drag. Nevertheless, these aspects need to be explored in greater depth and integrated to better understand their relationship and impact on performance.

### Kinematics

Swimming speed is determined by the product of stroke rate and stroke length [102]. The stroke rate has been related to neuromuscular power and energy capacities [103], while stroke length has been associated with force/strength and the ability to apply that force [53]. Both variables were positively associated with swimming performance, dry-land strength, and in-water force production [3, 42, 44, 49, 53, 55, 57, 58, 60, 63, 65, 67, 70, 72, 74]. Notwithstanding, the complex interaction between the two variables and their dependency on multiple factors (i.e. swimming stroke, technical skill level, physiological and muscular development [44, 53, 71, 72, 74, 103]) results in mixed and in parts contradictory effects of these two variables in a large number of studies [3, 42, 44, 49, 53, 55, 57, 58, 60, 63, 65, 67, 70, 72, 74]. However, it is clear that each swimmer should find the optimal combination of these parameters to improve performance [2, 60], which appears to be found at submaximal levels of both stroke rate and stroke length [60] and the difference lies in the capacity to increase one without negatively affecting the other [104].

Although not directly affected, both stroke rate and stroke length present a negative tendency when breathing during front crawl [44]. Swimmers tend to be slower overall when breathing since the inclusion of this action induces kinematic differences and likely kinetic asymmetries that affect the application of force during the strokes [47]. Kinematic differences observed in the ipsilateral side include lower shoulder flexion, abduction, and roll in the breathing trial during the entry phase, extended pull phase because of a shallower hand path, diminished shoulder abduction, slower hand vertical acceleration, and shortened push phase duration [44]. In general terms, a loss of 0.02–0.03 s per stroke cycle is estimated [44, 105], which, with such fine margins defining success, suggests that swimmers should control the number of breaths taken. In particular, in 50-m sprint events, the number of breaths should be reduced to none [94]. However, this result is from a single study conducted in front crawl and future research should be conducted to corroborate this fact and analyze the impact of breathing in butterfly.

In this review, only one study explored the effect of the initial speed after the start and turn on the subsequent swimming speed [66]. The results revealed the lack of influence of the initial speed (i.e., horizontal take-off velocity) in front crawl events [66]. However, there is a small difference in the transition phase (i.e., from the last underwater kick to the beginning of the stroke) that disappears as soon as the swimmers start stroking and a similar swimming speed is reached [66]. It is important to consider that from the initial speed in the work of Takeda et al. (2009) [66], the stroke speed was greater (in eight of the swimmers) than the transition speed. This lower speed during the transition not only evoked a lower performance (due to the momentaneous lower speed) but also a loss of energy, as swimmers need to accelerate during the first strokes until reaching the desired swimming speed [66].

Another distinguishing factor among sprinters is their peak speed, which has been shown to positively correlate with performance [71]. From biomechanical and energetic perspectives, it is more economical to swim at a constant speed than to have intra-cyclic speed variations. In this regard, research has revealed how proficient swimmers can adjust their coordination index at increasing speeds while maintaining a low and stable value of intra-cyclic speed variations [106]. However, in the case of sprint swimming, those swimmers that reached higher peak speed and stayed longer at the upper part of the speed curve were those that achieved higher performance [71]. Conversely, neither minimum speed nor intra-cyclic speed variations were associated with sprinters’ performance as both high- and low-level swimmers were able to reach similar minimum speed with differences in peak speed [71]. As a result, swimmers with a higher performance level showed higher intra-cyclic velocity variation compared with slower swimmers. Similar results were reported in elite breaststrokers, who presented higher intra-cyclic velocity variations than non-experts because of a combination of higher peak speeds with similar minimum speeds [107]. Hence, considering that intra-cyclic speed variation is associated with swimming efficiency [108, 109], these findings suggest that sprinters should prioritize training regimens that contribute to achieving and maintaining the highest possible speed rather than adopting the economical style typically found in middle- and long-distance [71].

### Anthropometrics

The anthropomorphological characteristics of swimmers have played an important role in the recent sprint swimming literature. Studies indicate that the fastest swimmers typically exhibit greater height, wider arm span, and larger body dimensions relative to their upper limbs and body mass [61, 77, 110]. The benefit of this higher dimension is attributed to the influence of body length on wave drag as greater height tends to decrease the Froude number, resulting in lower wave-making resistance [111]. Moreover, these effects are also mediated by body shape. For instance, torso morphology affects drag as the indentation at the waist and curvature of the buttocks may result in greater drag force and negatively affect swimming performance [112]. Hence, the interplay of these factors may be more intricate than expected, thereby complicating the relationship between anthropometric characteristics and [100] performance in all swimming strokes [61, 68].

Although the fastest swimmers tend to exhibit greater body dimensions [61, 77, 110], the anthropometric factors found to be predictive of performance varied considerably among strokes in females [68, 72, 77] and males, who also showed a lack of direct association [50, 55, 56, 68, 70, 72]. Although at first glance these results might seem contradictory, it is important to consider that the relationship between anthropometrics and performance might be mediated by other factors such as muscular strength or skill level [46, 60]. For instance, longer forearms may present a mechanical disadvantage, as they require the involved muscles to apply greater force and energy to overcome the drag associated with a longer length [29, 60, 113]. In this sense, Dopsaj et al. (2020) [61] found an association between front crawl performance and muscle mass in level 3 swimmers. Considering this performance level, it can be expected that swimmers had an excellent body position in the water and that these muscle masses were related to higher propulsion without a significant negative impact on drag. Moreover, considering the intricacy of certain strokes, such as butterfly, with respect to coordination, it is plausible that other factors may play a more substantial role in this context [55]. Future research should aim to study a homogeneous sample of high-level swimmers to further explore these associations while controlling for other factors that may influence these associations.

### Physiological Factors

In sprint swimming events, most of the energy is obtained via anaerobic pathways [114], with a clear domination of anaerobic carbohydrate catabolism [115]. In this regard, the research showed that higher [La^−^] seems to be related to higher swimming speeds [3, 58, 64, 69, 116]. Moreover, when the [La^−^] response was explored in depth, the results showed the importance of the [La^−^] reached but also its accumulation rate (denoted as VLa_max_), which has been positively associated with swimming speed [64]. Indeed, given the short duration of the effort, this parameter might be even more relevant. Despite the traditional belief that [La^−^] takes some minutes to reach its peak, level 1 swimmers reached extremely high values of [La^−^] (> 15 mmol/L) 30 s after ultra-short efforts (< 7 s) [117]. Such an extremely fast lactate production rate has also been shown in track and field athletes, with considerably higher [La^−^] values observed in top-level compared with sub-elite athletes [118]. These rapid responses following brief bouts of high-intensity efforts could be attributed to the activation of fast-twitch muscle fibers, which possess a range of metabolic profiles and robust power capabilities [119]. Although these results suggest that training should focus on both high [La^−^] values and VLa_max_, swimming research should explore in depth the lactate response across different performance levels and whether improvements in these metrics correlate with better sprint performance. It is important to note that [La^−^] is the balance between production and removal within the cell. Hence, these [La^−^] values may also increase by a reduction in the removal capability (associated with aerobic capabilities) [120]. Thus, although the aerobic pathway may play a less important role in sprint events (especially 50 m), it should be considered to develop a better understanding of the lactate response in sprint swimmers [121].

The aerobic component plays a less important role in sprint events than middle- or long-distance events and as a consequence of that, aerobic kinetics have not been highly explored in sprint events. However, its contribution can be as high as 50% in 100-m events [26, 122]. Indeed, only one study included in this review analyzed aerobic kinetics. The results revealed an association between aerobic kinetics and 100-m performance [69]. There was a positive association between performance and the amplitude of the fast component as well as a negative association with the time delay of the fast component. Indeed, both together accounted for 46% of the variance in 100-m performance, which suggests that swimmers should enhance their capacity to efficiently activate the aerobic system in addition to the anaerobic pathways to maximize the rate at which energy can be acquired [69]. Furthermore, pulmonary function may play an important role in these associations and respiratory muscle training was demonstrated to improve swimming performance [123]. In this context, forced inspiratory volume in the first second was highly associated with swimmers’ performance [50], which might be related to the limited time that each stroke allows the swimmer to inhale air [124], and the amount of oxygen inhaled per breath.

In contrast to findings in endurance athletes, Merati et al. [45] reported a positive correlation between vagal tone (NN50 and pNN50) and 50-m freestyle performance among sprinters. Specifically, lower vagal tone was associated with enhanced performance in the 50-m front crawl event. In that sense, the type of training impacts autonomic modulation. High-volume and low-intensity training periods typically result in parasympathetic predominance, while low-volume and high-intensity phases are associated with sympathetic predominance [125]. Hence, in this case, the type of training developed by sprinters likely induces a predominance of the sympathetic system, suggesting that the rapid suppression of cardiac vagal activity to elevate HR and enhance cardiac output is crucial for achieving optimal performance in sprint events, such as the 50-m front crawl [45]. Furthermore, post-training sympathetic activity showed a correlation with performance in the 100-m event, likely due to the heightened activation of the sympathetic nervous system in response to exercise during the preceding training session [45].

Training requires enough volume and intensity to develop the physiological parameters required to succeed [8]. For this reason, specific training sets are considered to develop these precise characteristics [126]. Terzi et al. (2021) [58] explored the suitability of a 4 × 50 m set (with 2 min of rest) to develop 100-m performance. They found that speed, lactate, SR and SI recorded during this test and 100 m were correlated with each other [58]. Therefore, the authors suggested this set as a suitable one to not only stimulate anaerobic metabolism but also to monitor 100-m performance. In this regard, it is important to note that swimmers evidenced higher speed, [La^−^], and stroke rate during the 4 × 50 m than during the 100-m test [58]. This might be seen as an appropriate stimulus to improve specific technical skills while dealing with related fatigue generated [11, 127].

### Limitations

The diverse methodologies and perspectives employed in sprint swimming literature, while expanding knowledge, also complicate our understanding of performance factors. While some domains (i.e., subsections) were thoroughly explored, others, such as the physiological aspects, were not. In this case, several of the findings discussed in this review are based on single studies, which limits their strength and highlights the need for further research. Many studies lacked a complete list of potential confounding factors on the study outcomes, which additionally complicates the interpretation of the results. Additionally, the lack of high-quality studies involving high-level swimmers (i.e., levels 1 and 2 [41]) further hampers the interpretation of the findings. Although these findings are of great interest to the overall swimming community, future research should focus on high-level samples to confirm the effects found at lower performance levels.

Regarding the methodological limitations, publications were limited to English, which may have caused relevant works on the subject to be missed. Although some of the authors are native speakers of other languages and could have accurately extracted the information, we made this decision to ensure that readers could re-read and understand all the research papers included in this systematic review. This has important implications for the verification of the results and further development in the field of research. Furthermore, three articles were excluded due to lack of access to the manuscripts. We contacted the authors multiple times via email, but had to exclude the articles due to no response. Finally, not including other databases such as conference proceedings databases may have precluded us from finding relevant studies on the subject.

## Conclusion

The current literature shows that sprint swimming performance depends on a wide variety of factors. Based on the findings, sprinters need to develop their muscular strength and properly transfer it to the water. However, it is important to note that the most effective way to accomplish this goal is still unclear. The velocity of the movement seems to be better related to performance than the load that can be displaced. This fact is of vital interest, as the whole dry-land training process may change considerably and need to be further investigated in the future, for example by comparing the effects of velocity and load-oriented trainings. Force application in the water is crucial; mastering the ability to apply the highest amount of force in a coordinated way (i.e., matching movements of the upper and lower limbs, together with an optimally streamlined body position) is key to achieving the highest speed. To do that, it is important to measure both propulsion and drag. Nevertheless, the majority of the studies focused on in-water forces or related aspects, leaving a small gap in the analysis of drag during maximal speed swimming.

Among the kinematic variables, stroke length and stroke rate play a crucial role in the development of better performance. These two parameters need to be optimally combined, together with other factors, but the intrinsic changes that occur with their modification are unknown. Hence, despite being widely explored, these parameters need to be explored in more depth. Swimmers’ anthropometry may play an important role in their performance; nevertheless, the interaction of these attributes appears to be intricate, suggesting that other factors may mediate or hold greater importance in determining performance outcomes.

It is important to highlight metabolic considerations for enhancing sprint performance. As such, swimmers should improve not only their lactate peak production but also its accumulation rate. A similar perspective might be considered in relation to the aerobic energy pathway. However, studies in this area are limited, and further research is needed to corroborate the existing evidence. The majority of the sprint-related research focused on front crawl, while the other strokes are significantly less explored. Indeed, some of the aspects applied to front crawl might be transferable to the others, but each stroke has its peculiarities and as such needs to be explored independently. Finally, the absence of a list of potential confounders, together with the lack of high-quality studies involving elite swimmers (level 1 and 2), complicates the interpretation of some results.

## Supplementary Information

Below is the link to the electronic supplementary material.

Supplementary file1 (DOCX 187 KB)

## Declarations

### Data Availability

The data supporting the findings of this study are available within the article and its supplementary materials.
