# Supplementary material for: Factors Relating to Sprint Swimming Performance: A Systematic Review
Source: Sports Med. 2025 Jan 22;55(4):899–922. doi: 10.1007/s40279-024-02172-4 (PMC12011652; doi:10.1007/s40279-024-02172-4)
Supplement: Supplementary file 1 — Supplementary file1 (DOCX 187 KB) [file 40279_2024_2172_MOESM1_ESM.docx]

**Electronic Supplementary Material**

**Factors Relating to Sprint Swimming Performance: A Systematic Review**

Jesús J. Ruiz-Navarro^1, *^, Catarina C. Santos^2,3^, Dennis-Peter Born^4,5,6^, Óscar López-Belmonte^1^, Francisco Cuenca-Fernández^1,7^, Ross H Sanders^8^, and Raúl Arellano^1^.

1. Aquatics lab, Department of Physical Education and Sports, Faculty of Sport Sciences, University of Granada, Granada, Spain.
2. Department of Sport Sciences, Higher Institute of Educational Sciences of the Douro (ISCE-Douro), Penafiel, Portugal
3. Higher Education School, Polytechnic of Coimbra, Coimbra, Portugal
4. Section for High-Performance Sports, Swiss Swimming Federation, Bern, Switzerland
5. Department for Elite Sport, Swiss Federal Institute of Sport Magglingen, Magglingen, Switzerland
6. Faculty of Science and Medicine, University of Fribourg, Fribourg, Switzerland
7. Department of Sports and Computer Sciences, Universidad Pablo de Olavide, Seville, Spain
8. Faculty of Medicine and Health, The University of Sydney, Sydney, Australia.

*** Corresponding author:**

Jesús J. Ruiz Navarro, PhD

Email: [jesusruiz@ugr.es](mailto:jesusruiz@ugr.es)

**ORCIDs:**

Jesús J. Ruiz-Navarro: <https://orcid.org/0000-0002-0010-7233>

Catarina C. Santos: <https://orcid.org/0000-0003-4592-8091>

Dennis-Peter Born: <https://orcid.org/0000-0002-1058-4367>

Óscar López-Belmonte: <https://orcid.org/0000-0003-4292-2460>

Francisco Cuenca-Fernández: <https://orcid.org/0000-0003-2942-4862>

Ross Sanders: <https://orcid.org/0000-0003-0489-3048>

Raúl Arellano: <https://orcid.org/0000-0002-6773-2359>

**Electronic Supplementary Material Table S1** Search terms used in Web of Science and Scopus databases.

| **Web of Science** |
| --- |
| TS=((((sprint) AND (swimming)) AND ((((((((((kinematics) OR (anthropometric)) OR (strength)) OR (biomechanics)) OR (physiology)) OR (race)) OR (lactate)) OR (training)) OR (propulsion)) OR (drag))) AND (performance)) |
| **Scopus** |
| TITLE-ABS-KEY ( ( ( ( sprint ) AND ( swimming ) ) AND ( ( ( ( ( ( ( ( ( ( kinematics ) OR ( anthropometric ) ) OR ( strength ) ) OR ( biomechanics ) ) OR ( physiology ) ) OR ( race ) ) OR ( lactate ) ) OR ( training ) ) OR ( propulsion ) ) OR ( drag ) ) ) AND ( performance ) ) |

**Electronic Supplementary Material Table S2** Adapted Downs and Black Quality Assessment Checklist

| **Reporting** | **Scores** |
| --- | --- |
| **1. Is the hypothesis/aim/objective of the study clearly described?** | 0 - 1 |
| **2. Are the main outcomes to be measured clearly described in the Introduction or Methods section?** If the main outcomes are first mentioned in the Results section, the question should be answered no. | 0 - 1 |
| **3. Are the characteristics of the participants included in the study clearly described?** In cohort studies and trials, inclusion and/or exclusion criteria should be given. In case‐control studies, a case‐definition and the source for controls should be given. | 0 - 1 |
| **5. Are the distributions of principal confounders in each group of subjects to be compared clearly described?** A list of principal confounders is provided | 0 - 2 |
| **6. Are the main findings of the study clearly described?** Simple outcome data (including denominators and numerators) should be reported for all major findings so that the reader can check the major analyses and conclusions. (This question does not cover statistical tests which are considered below). | 0 - 1 |
| **7. Does the study provide estimates of the random variability in the data for the main outcomes?** In non-normally distributed data the inter-quartile range of results should be reported. In normally distributed data the standard error, standard deviation or confidence intervals should be reported. If distribution data is not described, it must be assumed that the estimates used were appropriate and the question should be answered with yes. | 0 - 1 |
| **10. Have actual probability values been reported (e.g. 0.035 rather than <0.05) for the main outcomes except where the probability value is less than 0.001?** | 0 - 1 |
| **External validity** |  |
| **11. Were the subjects asked to participate in the study representative of the entire population from which they were recruited**? The study must identify the source population for subjects and describe how the subjects were selected. Subjects would be representative if they comprised the entire source population, an unselected sample of consecutive participants, or a random sample. Random sampling is only feasible where a list of all members of the relevant population exists. Where a study does not report the proportion of the source population from which the subjects are derived, the question should be answered as unable to determine. | 0 - 1 |
| **12. Were those subjects who were prepared to participate representative of the entire population from which they were recruited?** The proportion of those asked who agreed should be stated. Validation that the sample was representative would include demonstrating that the distribution of the main confounding factors was the same in the study sample and the source population. | 0 - 1 |
| **13. Were the staff, places, and facilities where the participants were treated, representative of the testing the majority of participants receive?** For the question to be answered yes, the study should demonstrate that the intervention was representative of that in use in the source population. The question should be answered no if, for example, the intervention was undertaken in a specialist centre unrepresentative of the hospitals most of the source population would attend. | 0 - 1 |
| **Internal Validity** |  |
| **16. If any of the results of the study were based on “data dredging”, was this made clear?** Any analyses that had not been planned at the outset of the study should be clearly indicated. If no retrospective unplanned subgroup analyses were reported, then answer yes. | 0 - 1 |
| **18. Were the statistical tests used to assess the main outcomes appropriate?** The statistical techniques used must be appropriate to the data. For example, nonparametric methods should be used for small sample sizes. Where little statistical analysis has been undertaken but where there is no evidence of bias, the question should be answered yes. If the distribution of the data (normal or not) is not described it must be assumed that the estimates used were appropriate and the question should be answered yes. | 0 - 1 |
| **20. Were the main outcome measures used accurate (valid and reliable)?** For studies where the outcome measures are clearly described, the question should be answered yes. For studies which refer to other work or that demonstrates the outcome measures are accurate, the question should be answered as yes. | 0 - 1 |
| **Internal Validity – confounding (selection bias)** |  |
| **21. Were the subjects in different intervention groups or were they recruited from the same population?** For example, participants for all comparison groups should be selected from the same hospital. The question should be answered unable to determine for cohort and case control studies where there is no information concerning the source of participants included in the study. | 0 - 1 |
| **Power** |  |
| **27. Did the study have sufficient power to detect a clinically important effect where the probability value for a difference being due to chance is less than 5%?** Sample sizes have been calculated to detect a difference of x% and y. | 0 - 1 |

**Electronic Supplementary Material Table S3** Quality assessment of the articles included

| References | 1 | 2 | 3 | 5 | 6 | 7 | 10 | 11 | 12 | 13 | 16 | 18 | 20 | 21 | 27 | Total | Total  score | Category |
| --- | --- | --- | --- | --- | --- | --- | --- | --- | --- | --- | --- | --- | --- | --- | --- | --- | --- | --- |
| Amara et al.[1] | 1 | 1 | 0 | 1 | 1 | 1 | 0 | 0 | 0 | 0 | 1 | 1 | 0 | 0 | 0 | 7 | 43.75% | Low |
| Barbosa et al.[2] | 1 | 1 | 1 | 0 | 1 | 1 | 1 | 0 | 0 | 0 | 1 | 1 | 1 | 0 | 0 | 9 | 56.25% | Good |
| Carvalho et al.[3] | 0 | 1 | 1 | 0 | 1 | 1 | 1 | 0 | 0 | 0 | 1 | 1 | 1 | 0 | 1 | 9 | 56.25% | Good |
| Chalkiadakis et al.[4] | 1 | 1 | 1 | 0 | 1 | 1 | 0 | 0 | 0 | 0 | 1 | 1 | 1 | 0 | 1 | 9 | 56.25% | Good |
| Dopsaj et al.[5] | 0 | 1 | 1 | 0 | 1 | 1 | 1 | 0 | 0 | 0 | 1 | 1 | 1 | 0 | 0 | 8 | 50.00% | Low |
| Gatta et al.[6] | 1 | 1 | 1 | 0 | 1 | 1 | 0 | 1 | 1 | 1 | 1 | 1 | 1 | 1 | 0 | 12 | 75.00% | Good |
| Gonjo et al.[7] | 1 | 1 | 1 | 0 | 1 | 1 | 1 | 0 | 0 | 0 | 1 | 1 | 1 | 0 | 0 | 9 | 56.25% | Good |
| Gonjo et al.[8] | 1 | 1 | 0 | 0 | 1 | 1 | 1 | 0 | 0 | 0 | 1 | 1 | 1 | 0 | 0 | 8 | 50.00% | Low |
| Gourgoulis et al.[9] | 1 | 1 | 0 | 0 | 1 | 1 | 1 | 0 | 0 | 0 | 1 | 1 | 1 | 0 | 0 | 8 | 50.00% | Low |
| Keiner et al.[10] | 1 | 1 | 1 | 0 | 1 | 1 | 1 | 0 | 0 | 0 | 1 | 0 | 1 | 0 | 0 | 8 | 50.00% | Low |
| Keiner et al.[11] | 0 | 1 | 1 | 0 | 1 | 1 | 0 | 0 | 0 | 0 | 1 | 1 | 0 | 0 | 0 | 6 | 37.50% | Low |
| Loturco et al.[12] | 1 | 1 | 1 | 0 | 1 | 1 | 0 | 0 | 0 | 0 | 1 | 1 | 1 | 0 | 0 | 8 | 50.00% | Low |
| Mavroudi et al.[13] | 0 | 1 | 0 | 0 | 1 | 1 | 1 | 0 | 0 | 1 | 1 | 1 | 1 | 0 | 0 | 8 | 50.00% | Low |
| McCabe et al.[14] | 1 | 1 | 0 | 0 | 1 | 1 | 1 | 0 | 0 | 0 | 1 | 1 | 1 | 0 | 0 | 8 | 50.00% | Low |
| Merati et al.[15] | 1 | 1 | 1 | 0 | 1 | 0 | 0 | 1 | 1 | 1 | 1 | 1 | 1 | 0 | 0 | 10 | 62.50% | Good |
| Morais et al. [16] | 1 | 1 | 1 | 0 | 1 | 1 | 0 | 0 | 0 | 0 | 1 | 1 | 1 | 0 | 1 | 9 | 56.25% | Good |
| Morais et al.[17] | 1 | 1 | 1 | 0 | 1 | 1 | 1 | 1 | 1 | 0 | 1 | 1 | 1 | 0 | 0 | 11 | 68.75% | Good |
| Morais et al.[18] | 1 | 1 | 1 | 0 | 1 | 1 | 1 | 1 | 1 | 0 | 1 | 1 | 1 | 0 | 0 | 11 | 68.75% | Good |
| Morouço et al.[19] | 0 | 1 | 1 | 0 | 1 | 1 | 1 | 0 | 0 | 0 | 1 | 1 | 1 | 0 | 0 | 8 | 50.00% | Low |
| Morouço et al.[20] | 1 | 1 | 0 | 0 | 1 | 1 | 0 | 1 | 1 | 1 | 1 | 1 | 1 | 0 | 0 | 10 | 62.50% | Good |
| Morouço et al.[21] | 1 | 1 | 1 | 0 | 1 | 1 | 0 | 0 | 0 | 0 | 1 | 1 | 1 | 0 | 0 | 8 | 50.00% | Low |
| Morouço et al.[22] | 1 | 1 | 0 | 0 | 1 | 1 | 1 | 0 | 0 | 0 | 1 | 1 | 1 | 0 | 0 | 8 | 50.00% | Low |
| Nicol et al. [23] | 1 | 1 | 1 | 1 | 1 | 1 | 0 | 0 | 0 | 0 | 1 | 1 | 1 | 0 | 0 | 9 | 56.25% | Good |
| Noriega-Sánchez  et al. [24] | 1 | 1 | 1 | 0 | 1 | 1 | 1 | 1 | 1 | 0 | 1 | 1 | 1 | 1 | 0 | 12 | 75.00% | Good |
| Özkadı et al.[25] | 1 | 1 | 1 | 0 | 1 | 1 | 1 | 0 | 0 | 0 | 1 | 1 | 1 | 1 | 0 | 10 | 62.50% | Good |
| Perez-Olea et al.[26] | 1 | 1 | 1 | 0 | 1 | 1 | 1 | 0 | 0 | 1 | 1 | 1 | 1 | 0 | 0 | 10 | 62.50% | Good |
| Rodriguez et al.[27] | 1 | 1 | 1 | 0 | 1 | 1 | 0 | 0 | 0 | 0 | 1 | 0 | 1 | 0 | 0 | 7 | 43.75% | Low |
| Rozi et al.[28] | 1 | 1 | 1 | 0 | 1 | 1 | 1 | 0 | 0 | 0 | 1 | 1 | 0 | 0 | 0 | 9 | 56.25% | Good |
| Ruiz-Navarro et al.[29] | 1 | 1 | 1 | 0 | 1 | 1 | 1 | 1 | 1 | 0 | 1 | 1 | 1 | 1 | 0 | 12 | 75.00% | Good |
| Ruiz-Navarro et al.[30] | 0 | 1 | 1 | 0 | 1 | 1 | 0 | 0 | 0 | 0 | 1 | 1 | 1 | 0 | 0 | 7 | 43.50% | Low |
| Schereven et al.[31] | 1 | 1 | 1 | 0 | 1 | 1 | 1 | 0 | 0 | 0 | 1 | 1 | 1 | 0 | 0 | 9 | 56.25% | Good |
| Siders et al.[32] | 0 | 1 | 0 | 1 | 1 | 1 | 0 | 0 | 0 | 0 | 1 | 1 | 1 | 1 | 0 | 8 | 50.00% | Low |
| Silva et al.[33] | 1 | 1 | 1 | 0 | 1 | 1 | 0 | 0 | 0 | 1 | 1 | 1 | 1 | 0 | 0 | 9 | 56.25% | Good |
| Simbaña-Escobarç  et al.[34] | 1 | 1 | 1 | 1 | 1 | 1 | 1 | 0 | 0 | 0 | 1 | 1 | 1 | 0 | 0 | 10 | 56.25% | Good |
| Strzała et al.[35] | 1 | 1 | 0 | 0 | 0 | 1 | 0 | 0 | 0 | 0 | 1 | 1 | 1 | 0 | 0 | 6 | 37.50% | Low |
| Strzała et al.[36] | 1 | 1 | 1 | 0 | 1 | 1 | 1 | 0 | 0 | 0 | 1 | 1 | 1 | 0 | 0 | 9 | 56.25% | Good |
| Strzała et al.[37] | 1 | 1 | 0 | 0 | 1 | 1 | 0 | 0 | 0 | 0 | 1 | 1 | 1 | 0 | 0 | 7 | 43.75% | Low |
| Takeda et al.[38] | 1 | 1 | 0 | 0 | 1 | 1 | 0 | 0 | 0 | 0 | 1 | 1 | 1 | 0 | 0 | 7 | 43.75% | Low |
| Terzi et al.[39] | 1 | 1 | 1 | 0 | 1 | 1 | 1 | 0 | 0 | 0 | 1 | 1 | 1 | 0 | 0 | 9 | 56.25% | Good |
| Criterion score (%) | 82.05 | 100.00 | 71.79 | 10.25 | 97.43 | 97.43 | 56.41 | 17.94 | 17.94 | 15.38 | 100.00 | 94.87 | 92.30 | 12.82 | 10.25 |  |  |  |
